# Cognitive impairment and pragmatics

**DOI:** 10.1186/s40064-016-1759-7

**Published:** 2016-02-19

**Authors:** Javier Gutiérrez-Rexach, Sara Schatz

**Affiliations:** College of Arts and Sciences, The Ohio State University, 1775 College Road, Columbus, OH USA

**Keywords:** Pragmatic competence, Illocutionary and perlocutionary function, Alzheimer’s disease

## Abstract

**Background:**

One of the most important ingredients of felicitous conversation exchanges is the adequate expression of illocutionary force and the achievement of perlocutionary effects, which can be considered essential to the functioning of pragmatic competence.

**Findings:**

The breakdown of illocutionary and perlocutionary functions is one of the most prominent external features of cognitive impairment in Alzheimer’s Disease, with devastating psychological and social consequences for patients, their family and caregivers.

**Conclusions:**

The study of pragmatic functions is essential for a proper understanding of the linguistic and communicative aspects of Alzheimer’s disease.

## Background

The inception of and most significant developments in pragmatic theory during the last century were firmly grounded in philosophical and linguistic pursuits. The study of linguistic utterances and their association with the speaker and addressee’s intentions, plans and beliefs has made significant contributions to contemporary philosophy of language and linguistic pragmatics. More recently, cognitive and social concerns have been brought to the fore with the goal of addressing the underlying psychological and social motivations explaining linguistic behavior and also the consequences of such behavior in these domains. The development of experimental pragmatics has also increased the connection with empirical research and scientific methodologies. This expansion of the field is leading to a reconceptualization of the basic concepts and hypotheses of pragmatic theory.

In the cognitive realm, the study of pragmatic competence and its evolution over the life span brings to the fore important issues such as the determination of how pragmatic components are acquired and how they decay; if they do so in a consistent sequence and the implications of such a sequence, etc. The study of dementia, and the several variants of Alzheimer’s Disease (AD), may constitute a privileged field to test the decay or preservation of pragmatic competence (Wray 2015a, b). It is also an ideal testing ground to witness the interplay of linguistic behavior with other interactional components in a more general cognitive and social environment, since dementia not only significantly affects the patients’ cognitive and pragmatic abilities but also has measurable effects on their relatives and other individuals socially or professionally involved with them. We argue for a reconceptualization of the landscape of cognitive decline and preservation in dementia that reinforces the validity of pragmatic theory as an important component of a general model of the cognitive and social dynamics underlying this disease.

## Models of global cognitive and linguistic decline

The literature on Alzheimer’s Disease (AD) emphasizes the centrality of progressive deficits in memory and other aspects of cognition as the key diagnostic criterion of the disease (DSM-IV-TR, American Psychiatric Association 2000; Reisberg et al. 1982; Helkala et al. 1988). In an effort to conceptualize how progressive impairment proceeds in the illness, cognitive impairment is a central feature of academic models of such disease. For example, Geldmacher’s (2012:130) model of impairment in AD refers to “domains of cognitive impairment”. In this model, “memory” is conceptualized as deficits in learning, semantic knowledge failure, repetitiveness; “executive functioning” is viewed as poor planning, poor judgment, impairment on complex tasks, disinhibition; “orientation” is understood in terms of distorted time sense, “praxis” is defined as ideomotor apraxia, limb-kenetic apraxia; “visual processing” as poor object or person recognition, spatial confusion, impaired directed attention. The definition of “language impairment” is restricted to anomia and difficult word-finding, poor speech content, impaired prosody (Geldmacher 2012:130). A diagnosis of AD requires memory impairment and impairment in at least one other “cognitive domain” (Geldmacher 2012:129). Such models are based on the idea that deficits resulting from synaptic dysfunction and neuronal loss follow a predictable distribution in the brain (Arab et al. 2011) and will result in the total global cognitive decline and total dependence of the AD patient on others.

Language and language decline is sometimes discussed as a discrete, separate capacity of AD decline but it too has been generally treated in *global terms* as a model of predicting eventual total language impairment (Grand et al. 2011). For example, Blair et al. (2007:241) argue that at early Alzheimer’s, word finding difficulty and circumlocution in conversation are present. Yet, speech deteriorates with disease progression becoming “verbose and circuitous…empty and lacking meaningful content” (Appell et al. 1982). Vocabulary decreases and irrelevancies increase. It becomes difficult to maintain a topic (Mentis et al. 1995). Ultimately, progressive aphasia leads to eventual mutism.

There is also a small literature focusing exclusively on the linguistic aspects of decline in AD and its effect on social interaction. Nevertheless, this literature also posits a uni-linear model of global linguistic decline. According to Wray (2015b), language processing is undermined by damage to the language areas of the brain. NLP features (lexicon, syntax errors) are evident in the natural speech of even early Alzheimer’s patients, as AD persons produce syntactically poorer sentences, mention lesser number of ideas and words, produce redundant, less precise and informative discourse; there is also rare use of the modalizers, and pronouns miss their intended reference, which implies a loss of the semantic cohesion (Boyé et al. 2014:4). The progressive loss of ability to communicate begins with early AD language deficits (word substitutions, aborted phrases), then progresses to comprehension deficits, paraphasic errors, and semantic jargon in mid-to-late stage Alzheimer (Tappen et al. 2002:63). Semantic and lexical speech features are multiple and include stutters, self-corrections, incomplete sentences, a greater number of empty pauses and lesser number of non-empty pauses or a very high percentage of personal pronouns (Boyé et al. 2014). Speech becomes formulaic and the patient might produce appropriate utterances even when there is doubt about whether they are really meant (Wray 2013; Hamilton 1994).

Ultimately, the effect of this loss of linguistic functions has significant social and pragmatic effects; more specifically, social isolation from other speakers. This negative spiral downward is interactive. As language becomes compromised by “short term memory loss, distortions in perception, disturbed semantic representation and disorientation brought on by lost contextual information”, linguistic behavior changes (Wray 2015b). People with [early to mid-stage AD] will apply “their remaining linguistic and communicative resources to rescue the situation, developing strategies for avoiding, compensating for, and covering up their problems”. Nevertheless, as a result of “loss of confidence, depression, altered power relationships, the social construction of AD as an illness, and the discourse contexts in which people with AD find themselves, such as how carers speak to them” (Wray 2015b), declines may be exaggerated due to the individual’s awareness of the problem and resultant frustration, embarrassment, or anxiety, “leading to further withdrawal” (Cohen 1991).

This, in turn, often provokes a negative reaction in caregivers who, in responding to the AD patient’s communicative difficulties, feel discomfort which can then inhibit their attempts to communicate with the patient which further reduces opportunities for the AD sufferer’s meaningful interaction (Hendryx-Bedalov 1999). Comprehension problems and their attempted resolution become products of social interaction and reflect pragmatic impairment (Perkins 2007). According to Tappen et al. (2002:63): “One of the most tragic symptoms of Alzheimer’s disease (AD) is the progressive loss of ability to communicate”. This loss has obvious adverse consequences for the older individual and their loved ones. For the person with AD, sequelae include “isolation, depression, disturbed behavior, and decreased quality of life” (Zanetti et al. 1998). Caregiver stress due to low levels of real communication with the patient is endemic (Wray 2014). The reduction in the AD patient’s capacity for empathic concern (other-centered emotional responses) can also have a negative effect on caregivers and marital relationships especially in later stages of the disease (de Vugt et al. 2003).

Thus, previous models of the decline of cognitive and specifically linguistic capacities in the disease course of AD have all posited a global model of decline as deficits in each of these areas progress to the endpoint of total cognitive decline and mutism. Untreated AD is, as Schioth et al. (2012:7) note, a “devastating disease”. In this article we advocate a model that focuses on pragmatic competence and the loss and re-gaining of cognitive/pragmatic functions.

## Performatives and pragmatic function

One of the landmarks in the emergence of pragmatic theory, as we know it today, is Austin’s (1962) proposal on certain sentences, which he called *performatives*. They contrast with other sentences in that a performative utterance (the utterance of a linguistic performative expression) is associated to or intrinsically constitutes the performance of an action. There are three acts related to an utterance and Austin suggests that a speaker can simultaneously perform three acts in issuing an utterance: the *locutionary* act is the act *of* saying something meaningful (to which a truth value can be ascribed); the *illocutionary* act is the act performed *in* saying something, essentially the performative utterance (the act identified by an explicit performative verb); and the *perlocutionary* act or effect, which is the act performed *by*, or the effect emerged as a consequence of saying something.

The perlocutionary/illocutionary distinction has not received as much attention in the literature. Nevertheless, it seems a critical distinction when adopting a broader view of pragmatic interaction, namely not only one that pays attention to the linguistic utterances and their structural configurations but also to the overall communicative picture, including the speaker’s intentions, the effect of linguistics interaction on conversational participants, and the cognitive and social mechanism underlying them. Pragmatic competence includes knowledge about the constraints regulating such interaction patterns and, as any other type of linguistic competence, it is part of the cognitive endowment of human beings (by nature). At the same time, it is also subject to evolution, including learning at the first stages of life and impairment and deterioration associated with the aging process and as a byproduct of disease (Perkins 2007). In what follows we will talk about pragmatic functions (of the illocutionary or perlocutionary type) to refer to the set of inferential processes, strategies, arrangements, and constraints regulating illocutionary force and perlocutionary effects for speakers. These types of pragmatic functions can be partly seen as belonging to executive-function mechanisms in general. For example, McDonald (1999) considers pragmatic inference generation and executive function as similar processes, given that “increasing degrees of impairment in the executive system correspond to greater and greater impairment of inferential reasoning”. Additionally, both executive function and inference require simultaneous attention and the processing of multiple sources of information in parallel. Executive function is by definition inextricably associated with the various cognitive, linguistic and sensorimotor elements in the intrapersonal domain over which it exercises control.

## Preservation of pragmatic function

The idea of intra-nasal insulin as a therapy to improve the cognitive capacities and quality of life for Alzheimer (AD) sufferers, their caregivers and families has been around since 1989. It was first proposed as a noninvasive intranasal method for bypassing the blood–brain barrier (BBB) by William H. Frey II and later expanded for the specific use of intranasal insulin to target the brain to treat Alzheimer’s disease and other CNS disorders (Thorne et al. 1995; Chen et al. 1998, 2004). Now, in 2015, as an AD therapy, it is receiving more scientific and media attention because it has been demonstrated to be safe in multiple, double-blind clinical studies with minimal side-effects as substantiated by currently available MRI brain imaging data and positive cognitive testing results on over 100 AD patients published in several peer-reviewed journals (Craft et al. 2012:9; Schioth et al. 2012:8; Claxton et al. 2014). Currently, it is in stage III of FDA review with 240 patients (The Study of Nasal Insulin in the Fight Against Forgetfulness (SNIF 2015) (www.clinicaltrials.gov, identifier–NCT01767909).

Memory decline and language impairment are prominent findings in AD (Grand et al. 2011). Yet, as noted, it has *also* been established in multiple, double-blind clinical trials that the administration of intra-nasal insulin improves cognition and verbal working memory in Mild Cognitive Decline and early AD patients (Shemesh et al. 2012:374; Claxton et al 2014). Studies of Mild Cognitive Decline and AD patients using *short*-*term* intra-nasal insulin (4 months or less) showed significantly less decline in cognition as measured by Voice Onset Time, Delayed Story Recall Score, the Dementia Rating Severity Scale and the ADCS-ADL scale as compared to the placebo group (Reger et al. 2008). Even *less relative decline* was found on the ADAS-cog score relative to the placebo group after 3–4 months of intra-nasal insulin use (Reger et al. 2006, 2008; Craft et al. 2012:31–32). Intranasal insulin’s dispersion into the frontal cortex is also extremely important for language capacities as it is the hypothesized site of verbal working memory in humans (Petrides et al. 1993). Pioneering participant-observation research, monitoring twenty-two “compassionate usage” Mild Cognitive Impairment and AD patients who have been on long-term intra-nasal insulin usage (2 years+) have shown marked improvement on intra-nasal insulin (Kurve Technology 2013). At baseline, before intra-nasal insulin treatment began, these “compassion usage” patients usually display some symptoms of social and linguistic withdrawal, flattening of affect and irritability. The return of illocutionary and perlocutionary capacity by these AD patients was also immediately noticed by the wives and partners (HBO Documentary 2009).^1^ Thus, the effect even after several months on intra-nasal insulin therapy showed evidence of significantly improved perlocutionary effects on spouses and partners.

The emergence of perlocutionary effects also relates to illocutionary capacity: the ability to express and to look for an effect of that expression. The effect that a person might want to achieve thorough a specific communication interaction can include such effects as validating the other’s persons feelings, agreeing with another’s feelings, expressing empathy for the other person, expressing solidarity with another and/or even to reduce the amount of perceived psychic pain caused by a disappointment. Hsieh et al. (2013:180–181) argues that in AD, the ability to perceive another person’s emotional state, to respond emotionally and to take the perspective of the other has been found to decline with progress of the disease, with empathy loss correlated with deficits in emotion processing. The aid of recognizing facial expressions of emotions is assumed to have a role in facilitating feelings of empathy (Hsieh 2013:180).

AD represents a loss of pragmatic competence especially in the illocutionary and perlocutionary functions associated with interactive and expressive linguistic domains. Intra-nasal insulin treatment seems to preserve or improve pragmatic function related to illocutionary force and perlocutionary effect. All these aspects achieve significant pragmatic effects (strong, positive perlocutionary responses on the listeners), thereby reducing the potential social isolation of the Mild Cognitive Impairment or AD patient.

## Conclusions

Focusing on effects related to pragmatic competence suggests that the term “cognitive impairment” in AD should be understood as a term including not just processes and domains related mostly to memory (short/long-term recall) but also include perception, language, self-awareness and awareness of others. As Geldmacher (2012:128) acknowledges, the academic attempt to create a model of “cognitive” decline in AD (which subsumes language) is ultimately artificial because “parsing cognitive functions into specific domains reflects the conveniences of taxonomy and testing rather than physiological reality”. He notes that “intact human cognition is a seamless and interdependent whole”. Improvements in pragmatic competence can occur with extended intra-nasal insulin treatment *even if* other cognitive capacities such as memory do not necessarily improve or change.

## Footnotes

1 Research on the short-term administration of intra-nasal insulin (3–4 months) also suggests that cognitive functions be subsequently assessed individually after treatment in addition to examining performance on global cognitive indicators (Craft et al 2014:8). This is because different areas of the brain appear to have different dose response profiles (Craft et al 2014:8).
